# Epidural Spinal Cord Stimulation of Lumbosacral Networks Modulates Arterial Blood Pressure in Individuals With Spinal Cord Injury-Induced Cardiovascular Deficits

**DOI:** 10.3389/fphys.2018.00565

**Published:** 2018-05-18

**Authors:** Sevda C. Aslan, Bonnie E. Legg Ditterline, Michael C. Park, Claudia A. Angeli, Enrico Rejc, Yangsheng Chen, Alexander V. Ovechkin, Andrei Krassioukov, Susan J. Harkema

**Affiliations:** ^1^Department of Neurological Surgery, University of Louisville, Louisville, KY, United States; ^2^Kentucky Spinal Cord Injury Research Center, University of Louisville, Louisville, KY, United States; ^3^Department of Neurosurgery and Neurology, University of Minnesota School of Medicine, Minneapolis, MN, United States; ^4^Frazier Rehab Institute, Louisville, KY, United States; ^5^Experimental Medicine Program, University of British Columbia, Vancouver, BC, Canada; ^6^International Collaboration on Repair Discoveries, Division of Physical Medicine and Rehabilitation, Department of Medicine, University of British Columbia, Vancouver, BC, Canada; ^7^GF Strong Rehabilitation Centre, Vancouver Coastal Health, Vancouver, BC, Canada

**Keywords:** orthostatic hypotension, systemic hypotension, human spinal cord injury, epidural stimulation, blood pressure

## Abstract

Disruption of motor and autonomic pathways induced by spinal cord injury (SCI) often leads to persistent low arterial blood pressure and orthostatic intolerance. Spinal cord epidural stimulation (scES) has been shown to enable independent standing and voluntary movement in individuals with clinically motor complete SCI. In this study, we addressed whether scES configured to activate motor lumbosacral networks can also modulate arterial blood pressure by assessing continuous, beat-by-beat blood pressure and lower extremity electromyography during supine and standing in seven individuals with C5-T4 SCI. In three research participants with arterial hypotension, orthostatic intolerance, and low levels of circulating catecholamines (group 1), scES applied while supine and standing resulted in increased arterial blood pressure. In four research participants without evidence of arterial hypotension or orthostatic intolerance and normative circulating catecholamines (group 2), scES did not induce significant increases in arterial blood pressure. During scES, there were no significant differences in electromyographic (EMG) activity between group 1 and group 2. In group 1, during standing assisted by scES, blood pressure was maintained at 119/72 ± 7/14 mmHg (mean ± SD) compared with 70/45 ± 5/7 mmHg without scES. In group 2 there were no arterial blood pressure changes during standing with or without scES. These findings demonstrate that scES configured to facilitate motor function can acutely increase arterial blood pressure in individuals with SCI-induced cardiovascular deficits.

## Introduction

Spinal cord injury (SCI) can be catastrophic with significant health and financial implications for patients, their families, and society (Ma et al., 2014). In addition to motor and sensory impairment, SCI is commonly associated with cardiovascular dysfunction, one of the leading causes of death in the SCI population (Chopra et al., 2016; Wecht and Bauman, 2017). Impairment to the sympathetic pathways can result in poor cardiovascular regulation, leading to persistent resting and orthostatic hypotension (Furlan et al., 2003). Persons with SCI often report symptoms of orthostatic intolerance during and/or right after the therapeutic interventions (Illman et al., 2000). These impairments are severe enough to delay hospital discharge, restrict an individual's participation in rehabilitation, and delay the achievement of functional goals (Blackmer, 1997; Claydon et al., 2006). Additionally, individuals with SCI report symptoms of blood pressure dysregulation cause them to avoid social situations and limits their independence (Carlozzi et al., 2013).

Current management approaches for systemic and orthostatic hypotension in SCI include pharmacological and non-pharmacological options (Krassioukov et al., 2009). Pharmacological interventions have limitations (e.g., presence of sustained hypertension and potential to exacerbate episodes of autonomic dysreflexia) (Teasell et al., 2000; Nieshoff et al., 2004; Freeman, 2008; Wecht et al., 2010), while non-pharmacological treatments and therapeutic interventions have demonstrated little success at ameliorating systemic hypotension in the long-term despite hemodynamic changes that may occur acutely (Faghri and Yount, 2002; Gillis et al., 2008; Mills et al., 2015). Essentially, those with chronic SCI have few options to maintain adequate systemic blood pressure which severely limits application of other therapies (Blackmer, 1997; Illman et al., 2000; Carlozzi et al., 2013).

We have previously demonstrated that spinal cord epidural stimulation (scES) at the lumbosacral level enabled four individuals with chronic, motor complete SCI to stand and generate voluntary movement in their lower limbs (Harkema et al., 2011; Angeli et al., 2014; Rejc et al., 2015, 2017a). This finding indicates scES can activate motor networks below the level of injury. Additionally, it has been shown that electrical stimulation of lower limb muscles can increase blood pressure and heart rate in an upright position and in response to orthostatic stress in individuals with complete SCI (Elokda et al., 2000; Sampson et al., 2000; Faghri and Yount, 2002; Jacobs et al., 2003; Chao and Cheing, 2005). Epidural stimulation of the lumbosacral spinal cord can increase peripheral blood flow and blood pressure in neurally intact individuals (Huber et al., 2000; Yamasaki et al., 2006) and maintain normative blood pressures in individuals with motor complete SCI (Harkema et al., 2018). This study investigates acute cardiovascular effects of scES utilized specifically to facilitate motor activity in persons with SCI without extensive locomotor training. We hypothesized that scES used to facilitate motor activity would also modulate cardiovascular function in persons with SCI.

## Materials and methods

### Research participants

Seven males, 26.7 ± 4.1 years of age, with chronic C5-T4 SCI participated in this study from 2009 to 2015. According to the International Standards for the Neurological Classification of Spinal Cord Injury (ISNCSCI), the neurological level and completeness of the spinal cord lesion were determined using the American Spinal Cord Injury Association Impairment Scale (AIS) (Kirshblum et al., 2011). Individuals B07, B13, and B23 (neurological levels of injury from C5 to T2) demonstrated impaired sensory and no motor function below the level of injury (AIS B). Individuals A45, A53, A59, and A60 were classified as AIS A, demonstrated neither sensory nor motor function below the neurological lesion at T4 (Table 1). Inclusion criteria: (1) stable medical condition without cardiopulmonary disease or dysautonomia that would contraindicate standing or stepping with BWST; (2) no painful musculoskeletal dysfunction, unhealed fracture, contracture, pressure sore, or urinary tract infection that might interfere with stand or step training; (3) no clinically significant depression or ongoing drug abuse; (4) no current anti-spasticity medication regimen; (5) non-progressive SCI above T10; (6) must not have received botox injections in the prior 6 months; (7) be unable to stand and step independently overground; (8) unable to voluntarily move individual joints of the legs; (9) no descending volitional control of movement below the lesion detected by neurophysiological testing; (10) segmental reflexes remain functional below the lesion; (11) at least 1-year post injury; and (12) must be at least 18 years of age. All procedures and assessments were carried out after informed consent was obtained as approved by the University of Louisville (KY, USA) and the University of California, Los Angeles (CA, USA) Institutional Review Boards in accordance with the Declaration of Helsinki.

**Table 1:** Characteristics of Spinal Cord Injured (SCI) Participants.

							**AIS score**					
							**Motor**		**Sensory**			
									**Light touch**		**Pinprick**	
	**Participant**	**Post injury (Year)**	**Height (cm)**	**Weight (kg)**	**Neuro level**	**AIS grade**	**C5-T1 (L/R)**	**L2-S1 (L/R)**	**C2-T4 (L/R)**	**T5-S5 (L/R)**	**C2-T4 (L/R)**	**T5-S5 (L/R)**
Group 1	B23	3.4	188	73	C5	B	12/11	0/0	16/15	13/15	12/12	0/0
	B13	3.5	180	97	C7	B	24/20	0/0	22/22	26/24	18/18	10/11
	B07	2.8	185	98	T2	B	25/25	0/0	22/22	28/24	18/18	10/11
Group 2	A60	2.7	188	86	T4	A	25/25	0/0	22/22	2/2	22/20	0/0
	A59	2.3	185	66	T4	A	25/25	0/0	22/22	1/1	22/22	1/1
	A53	2.3	178	64	T4	A	25/25	0/0	22/22	2/2	22/22	0/1
	A45	2.0	183	102	T4	A	25/25	0/0	22/22	2/0	22/22	0/0

*Neuro Level, Neurological level of injury; AIS, American spinal injury assessment Impairment Scale; L, left; R, right. All participants were males, 29 ± 5.2 (Group 1) and 25 ± 2.6 (Group 2) years of age*.

### Surgical implantation of electrode array and stimulator

**Figure 1:**
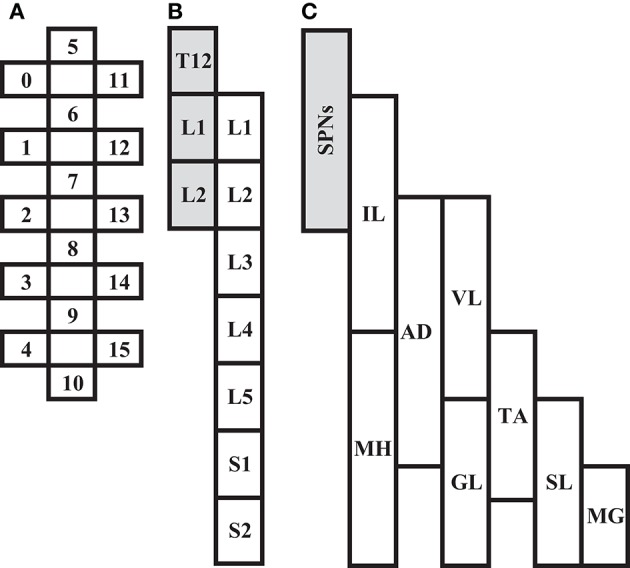
Depiction of scES 16-electrode array **(A)** relative to spinal cord segments L1 to S2 **(B)**, and corresponding muscles (**C**; IL, Iliopsoas; MH, Medial Hamstrings, AD, Adductor Magnus; VL, Vastus Lateralis; GL, Gluteus Maximus; TA, Tibialis Anterior; SL, Soleus; MG, Medial Gastrocnemius). Shaded areas **(B,C)** represent localization of spinal sympathetic preganglionic neurons (SPNs) at T12-L2 levels.

In all participants, an epidural spinal cord stimulator unit (RestoreADVANCED, Medtronic, Minneapolis, MN, USA) in combination with a 16-electrode array (Specify 5-6-5, Medtronic, Minneapolis, MN, USA) were implanted at the T11-L1 vertebral levels over the spinal cord segments L1-S1 as previously described (Harkema et al., 2011; Angeli et al., 2014). The stimulator and electrode array were used to deliver electrical stimulation to the lumbosacral enlargement of the spinal cord during selected assessments. A graphical representation and placement of the electrode array relative to spinal cord segments and peripheral nervous system (somatic and autonomic) are illustrated in Figure 1.

### Data acquisition

#### Orthostatic stress test

All participants were assessed for orthostatic tolerance prior to scES implantation. All studies were performed in the morning in a quiet, temperature controlled (~22°C) laboratory. Participants were instructed to avoid caffeine, alcohol, and high-fat foods for 12 h prior to their examination. Beat-to-beat blood pressure from a finger cuff (Portapres Model-2, Finapres Medical System, Amsterdam, Netherlands), calibrated to brachial blood pressure measurements (Dinamap V100 BP, General Electric Healthcare, Dallas, TX) (Bos et al., 1996), and a single lead electrocardiogram (ECG, lead II; ML132, ADInstruments) were acquired at 1,000 Hz using ML880 PowerLab 16/30 system. Serum catecholamines were obtained from a blood sample drawn from a butterfly catheter inserted into an antecubital vein prior to the test. Participants began the study in the supine position for 15 min, after which they were moved rapidly to a seated position for an additional 15 min. Blood was drawn in the supine position just prior to the sitting transition (within 3 min), and at approximately minutes 3 and 10 of the sitting position in order to measure resting serum catecholamines levels during acute and prolonged phases of the orthostatic stress test.

#### Hemodynamic and motor activity in response to scES

Beat-by-beat blood pressure, ECG, and electromyography (EMG) were recorded continuously, as previously described, while the individual was supine or standing over ground (Rejc et al., 2015); supine and standing experiments were not performed on the same day. EMG signals were recorded bilaterally using MA300 EMG System (Motion Lab Systems, Baton Rouge, LA) with bipolar surface electrodes placed longitudinally on the medial hamstrings, adductor magnus, vastus lateralis, gluteus maximus, tibialis anterior, soleus, and medial gastrocnemius muscles. Inter-electrode distance was fixed at 17 mm, center-to-center. EMG from iliopsoas muscles was recorded with fine-wire electrodes. To detect stimulation artifact, two surface electrodes were placed symmetrically over the paraspinal muscles, lateral to the electrode array incision. EMG data were collected at 2,000 Hz using a custom acquisition software (National Instruments, Austin, TX). EMG signals were differentially amplified with a band-pass filter of 10 Hz−2 kHz (−3 dB). Supine experiments assessed EMG and cardiovascular response to rostral and caudal configuration of the electrode array. Stimulation was maintained at a constant frequency of 2 Hz, while amplitude increased from 0 to 10 V, or the maximum voltage tolerated by the individual. Voltage was increased by 0.1 V intervals until all muscles (listed previously) showed a motor evoked response, and then increased by 0.5 V intervals thereafter. In standing experiments, voltage, frequency, and configuration of the electrode array were unique to each participant and optimized for over-ground standing. Data were obtained from 3 min of continuous blood pressure recordings after the individual had completed the transition from sit to stand, and the voltage had reached the level to sustain stable standing.

### Data analysis

Blood pressure and heart rate analyses were performed off line using Matlab (The MathWorks) software. The locations of the R waves in the ECG were identified to construct beat-to-beat heart rate and RR interval time series. The maximum and minimum values of blood pressure between two RR intervals were computed as beat-by-beat sampled systolic and diastolic blood pressure. Group hemodynamic variables (mean ± SD) during orthostatic stress test were calculated for 1-min intervals in the sitting position. Values obtained in the seated position were compared with the last 5 min of supine by calculating the difference. Data recorded during the initial 15 s of the sitting position were excluded from analysis because of movement artifacts. In supine experiments, means of blood pressure and heart rate data were calculated for periods during each 1 V increment; data are reported as percentage-change from baseline to normalize hemodynamic response to stimulation between participants. During standing experiments, blood pressure and heart rate were obtained continuously. Blood pressure and heart rate were averaged during sitting and standing during 3 consecutive minutes.

EMG amplitude of motor evoked responses to rostral and caudal scES was quantified by peak to peak amplitude (Rejc et al., 2015). The mean of five peak to peak amplitudes at each stimulation intensity, ranging from 0.1 V to 10 V, were used to quantify EMG activity by intensity. Group EMG activity (mean ± SD) at each stimulation intensity were used for between-group comparisons. All analyses were performed with customized software in MATLAB (Mathworks, Natick, MA, USA).

### Statistical analysis

We fit generalized additive models to examine if there were significant differences between participants in cardiovascular response to the orthostatic stress test. The models of cardiovascular parameters (systolic and diastolic blood pressure and heart rate) included factors accounting for group and time (the primary covariate). The models of EMG activity investigated differences in EMG activity between groups during the supine experiment. Stimulation voltage was the primary covariate, with factors accounting for configuration (rostral vs. caudal) and group (i.e., cardiovascular response to the orthostatic stress test) with an interaction between them. Smoothing spline functions were included in each model, defined as functions of the primary covariate (time for the orthostatic stress test models, stimulation voltage for the EMG activity models). We used a two-way, repeated measures ANOVA to test for significant between-group differences in blood pressure with and without scES, and catecholamines at rest and in response to orthostatic stress. For each model, an interaction between group and position was included. The models were fit using the open-source R software environment (R: A language and environment for statistical computing, R Foundation for Statistical Computing, Vienna, Austria). Significance was set to α < 0.05.

## Results

### Orthostatic stress test and blood catecholamines

**Figure 2:**
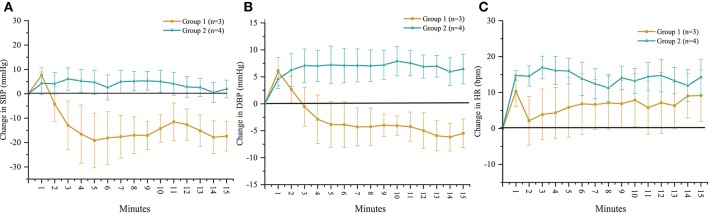
Time course of change in **(A)** systolic blood pressure (SBP), **(B)** diastolic blood pressure (DBP) and **(C)** heart rate (HR) in response to orthostatic stress test performed without scES. Group 1 (*n* = 3) SBP (*p* < 0.001), DBP (*p* < 0.001), and HR (*p* < 0.01) changed significantly compared with baseline; group 2 (*n* = 4) demonstrated no significant changes to SBP, DBP, or HR from baseline. Data are represented as mean ± SD.

**Figure 3:**
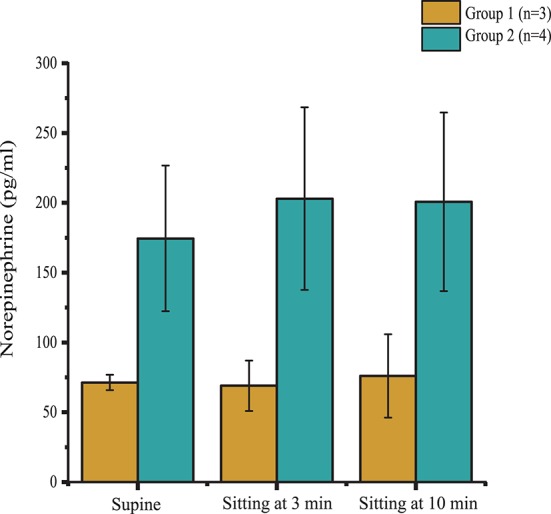
Plasma norepinephrine levels in supine position, and during minutes 3 and 10 of orthostatic stress. Norepinephrine levels were significantly lower (*p* = 0.02) in Group 1 (*n* = 3) compared with Group 2 (*n* = 4) throughout the orthostatic stress test. Data are represented as mean ± SD.

According to the outcomes obtained during the orthostatic stress test, participants were divided into two groups (Table 1). Compared with group 2, participants in group 1 experienced a significant change in systolic blood pressure (*p* < 0.001), diastolic blood pressure (*p* < 0.001), and heart rate (*p* < 0.01) during the orthostatic stress test (Figure 2) compared with baseline; participants in group 1 had significantly (*p* = 0.02) lower circulating catecholamines (Figure 3), when compared with group 2, which were within normal ranges (Yamanouchi et al., 1998). Individuals in group 2 demonstrated no evidence of orthostatic intolerance (Figure 2 and Table 2). During orthostatic stress, catecholamines did not increase significantly compared with supine levels in either group (Figure 3).

**Table 2:** Blood pressure and heart rate in sitting position without scES and during standing with and without scES.

		**Blood pressure and heart rate without scES**				**Blood pressure and heart rate with scES**				
	**Participant**	**Sitting**		**Standing**		**Standing**				
		**mmHg**	**BPM**	**mmHg**	**BPM**	**mmHg**	**BPM**	**Voltage**	**Hz**	**Configuration**
Group 1	B23	112/75	65	66/54	98	112/80	84	5.0	30	7– 10– 13– // 2+ 4+ 15+
	B13	92/60	80	76/43	99	119/55	133	5.0	15	4– 10– 15– // 9+
	B07	107/69	90	70/40	116	126/80	134	7.5	15	4– 10– 15– // 3+ 9+ 14+
Group 2	A60	107/56	54	94/62	89	107/65	91	2.5	25	4– 10– 14– 15– // 3+ 12+
	A59	115/62	80	112/65	67	108/64	80	2.4	25	4– 10– // 6+ 12+
	A53	118/74	76	117/79	90	118/83	92	2.7	35	4– 10– 15– // 3+ 8+ 14+
	A45	123/69	93	128/85	81	119/77	95	4.8	25	4– 10– 14– // 3+

*Note that with scES “off”, participants in Group 1 demonstrated significant drop in systolic and diastolic blood pressure upon standing (p = 0.008 and p = 0.01, respectively). Note also that systolic and diastolic blood pressure values when scES was “on” in standing position were not significantly different between groups; participants in group 2 demonstrated neither a drop in blood pressure upon standing nor change in blood pressure when stimulation was applied*.

### Blood pressure and EMG responses to scES in the supine position

**Figure 4:**
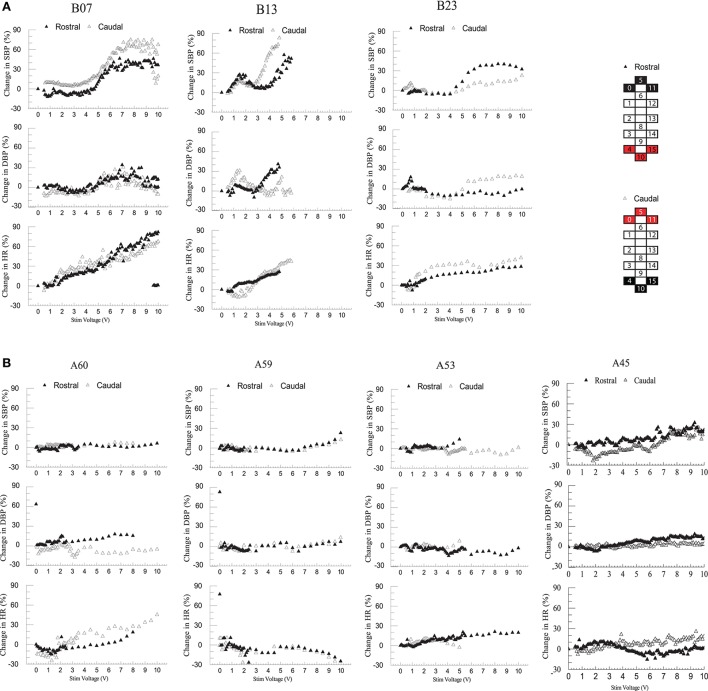
Effect of rostral (0–5–11–/4+10+15+) and caudal (4–10–15–/0+5+11+) scES at 2 Hz on supine blood pressure and heart rate. Illustrated from group 1 (**A**; *n* = 3) and group 2 (**B**; *n* = 4) are mean percent-change in systolic blood pressure (SBP), diastolic blood pressure (DBP), and heart rate (HR) from baseline (open triangles: rostral; black triangles: caudal stimulations) concurrent with increases in stimulator voltage. Supine voltage increased from 0 V to 10 V by 0.1 V and 0.5 V intervals. Electrode configuration and color map are presented on the right side of the figure; black boxes are cathode, red boxes are anode, and white boxes are inactive electrodes.

**Figure 5:**
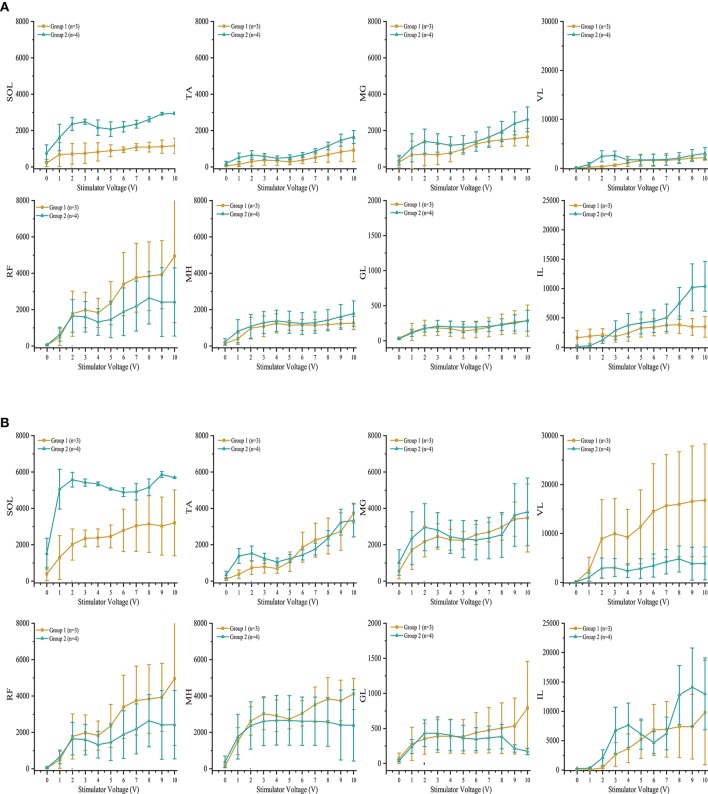
Effect of scES at 2 Hz with **(A)** rostral (0–5–11–/4+10+15+) and **(B)** caudal (4–10–15–/0+5+11+), stimulation configurations on muscle activity. Illustrated is mean electromyography (EMG) of leg muscles (SOL, Soleus; TA, Tibialis Anterior; MG, Medial Gastrocnemius; VL, Vastus Lateralis; RF, Rectus Femoris; MH, Medial Hamstrings; GL, Gluteus Maximus; IL, Iliopsoas) from group 1 (gold circle; *n* = 3) and group 2 (green triangle, *n* = 4) as simulator voltage increased from 0 V to 10 V by 0.1 V and 0.5 V intervals. There were no significant differences in EMG activity between groups during rostral and caudal scES configurations. Data are represented as mean ± SD.

Individuals in group 1 demonstrated increases in systolic blood pressure, diastolic blood pressure, and heart rate in response to increases in voltage of rostral and caudal scES configurations (Figure 4A). In contrast, individuals in group 2 demonstrated no increase in blood pressure regardless of stimulation voltages or polarity (Figure 4B); heart rate increased only in A60. During supine stimulation, all participants presented similar increases in EMG activity of leg muscles (Figures 5A,B; *p* > 0.05): regardless of configuration of the electrode array, each muscle demonstrated a gradual increase in EMG activity with increasing stimulator voltage (Figures 5A,B).

### Blood pressure responses during standing with and without scES

**Figure 6:**
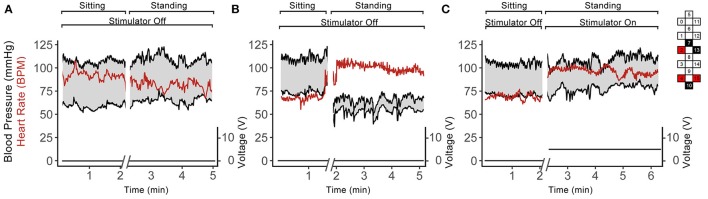
Continuous blood pressure and heart rate recordings from A59 **(A)** and B23 **(B,C)** in sitting and standing positions while participant was sitting and standing without scES **(A,B)** and using scES **(C)**. Top and bottom black lines indicate systolic and diastolic blood pressure; red line indicates heart rate. The stimulator intensity and electrode configuration are given on the right side; black boxes are cathode, red boxes are anode, and white boxes are inactive electrodes. The stimulation frequency was 15 Hz. Note that: subject A59 did not experience a drop in blood pressure upon standing **(A)**, while subject B23 experienced such drop **(B)**, and this decrease in blood pressure was abolished in the presence of the stimulation.

Similar to the orthostatic stress test, individuals in group 1 experienced profound drops in arterial blood pressure upon standing (Table 2). These participants reported feeling dizzy or fatigued such that they could not continue standing. When the stimulator was on, the drop in blood pressure and orthostatic symptoms were ameliorated (Table 2). Participants in group 2 did not experience a drop in arterial blood pressure upon standing, and application of scES did not increase their blood pressure further (Table 2). Figure 6 illustrates representative blood pressure recordings from group 2 (A59) and group 1 (B23) during standing without scES (Figures 6A,B) and with scES (Figure 6C). Representative of group 2, A59 was able to maintain his blood pressure while standing. However, B23, representative of group 1, demonstrated a blood pressure decrease to 64/54 mmHg while standing, compared with 112/75 mmHg while sitting. With application of scES, B23 stood upright with minimal assistance from trainers and maintained his blood pressure at a mean of 112/80 mmHg (Figure 6).

## Discussion

We demonstrated that lumbosacral scES configured to facilitate lower limb motor activity can modulate blood pressure in SCI individuals with detectable cardiovascular deficits. Increase in scES voltage increased EMG activity of leg muscles in all participants, but modulation of arterial blood pressure was observed only in participants with cardiovascular dysregulation. When used to facilitate standing, scES prevented a significant drop in blood pressure in participants with cardiovascular dysregulation, but had no effect on blood pressure regulation in participants that demonstrated no cardiovascular deficits.

Reduction in sympathetic drive below the level of SCI and cardiovascular deconditioning from inactivity are two potential major contributors to of cardiovascular dysfunction after SCI (Wecht and Bauman, 2017; Draghici and Taylor, 2018). Descending sympathetic pathways provide tonic control to sympathetic preganglionic neurons located in the spinal cord, which synapse on postganglionic neurons that emerge from levels T1 to T5 to modulate heart rate, from T5 to T11 to modulate catecholamine release, and from T1 to L2 to maintain vasomotor tone; the degree of sympathetic impairment thus correlates strongly with resting blood pressure and plasma catecholamine levels after SCI (Furlan et al., 2003). Level of injury of participants B07, B13, and B23, i.e., T2 or higher, corresponds to the loss of the majority of sympathetic outflow to the heart, blood vessels, and adrenal medulla, confirmed by decreased circulating catecholamines and blood pressure instability while upright (Figures 2, 3, 6). The inability to maintain blood pressure during orthostatic stress in group 1 could be attributed to a lack of sympathetic modulation as well as a deconditioned baroreflex, thereby limiting heart rate response (Wecht et al., 2006; Draghici and Taylor, 2018). Level of injury of the remaining participants was T4; this may result in the preservation of some cardiac sympathetic control, and some modulation of medullary control and vasomotor tone. Normally, increased venous return, such as from skeletal muscle contraction, has little effect on arterial blood pressure because changes in stroke volume are rapidly compensated by heart rate (Stead and Warren, 1947; Triedman and Saul, 1994). Differences in spinal sympathetic outflow and baroreflex response between groups could possibly be why scES increases blood pressure while supine in group 1 but not group 2 (Figure 4). Participants with better preserved autonomic control would have more regulatory mechanisms available to compensate for neuromuscular blood redistribution in response to scES, thus buffering the increased venous return into the circulation with minimal changes to blood pressure. On the other hand, alleviation of orthostatic hypotension in group 1 suggests scES may have a beneficial effect during weight bearing motor activity at higher frequencies since blood pressure remains within normal ranges and orthostatic hypotension is mitigated (Figure 6). This is consistent with our recent findings that acutely stimulating the lumbosacral spinal cord with configurations targeted to elevate blood pressure without muscle activation can ameliorate hypotension in individuals with SCI (Harkema et al., 2018).

In the present study, participants in both groups demonstrated similar increases in EMG activity of leg muscles in response to increasing scES voltage when evaluating both rostral and caudal configurations of the electrode array. We have found previously that configuration of the electrode array is a crucial determinant of individual muscle activation. When effects of scES were examined on both cardiovascular outcomes and EMG activity of leg muscles, we found that blood pressure increased only in those individuals who had cardiovascular deficits. Increased EMG activity combined with increased blood pressure suggests activation of the skeletal muscle pump and increased venous return (Miller et al., 2005). The increase while supine was varied: blood pressure did not change when voltage was below 4 V in B13 and B23, and below 5 V in B07 but increased up to 90% above initial supine values as voltage increased (Figure 4A). There was no further increase in blood pressure after 6 V in B07 and B23. The dramatically increased blood pressure during supine stimulation may be a result of increasing strength of muscle contractions (Miller et al., 2005), no redistribution of blood volume to the lower extremities from gravity (Freeman et al., 2011), and possible activation of residual sympathetic fibers below the level of injury in response to scES (Stauss et al., 1997).

In animal models, cats demonstrate increased both sympathetic nerve activity and mean blood pressure in response to scES at T12-L1 (Yanagiya et al., 2004), while stimulation of the lumbar sympathetic trunk in rats leads to increased vasoconstriction and increased mean arterial blood pressure (Gillespie and Muir, 1967; Stauss et al., 1997). Our findings taken in the context of animal studies suggest that blood pressure increase subsequent to scES may not be only neuromuscular in nature, i.e., increased venous return from skeletal muscle contraction. While we did not attempt to selectively locate and activate sympathetic lumbar networks with scES, nor did we measure diameter of the vessels in the lower limbs, it is still possible that scES was activating spinal networks responsible for modulation of cardiovascular function. Due to proximity of the implant, the electrical current could activate caudal sympathetic circuits of the spinal cord (T12-L2, Figure 1) or neighboring interneurons, exciting spinal sympathetic circuits to elevate arterial blood pressure. Interestingly, however, there were no significant changes in blood pressure when comparing rostral with caudal stimulation configurations. Due to proximity of the rostral electrodes to sympathetic preganglionic neurons, we hypothesized rostral stimulation configurations would generate greater increases in blood pressure due to proximity of sympathetic vasomotor neurons. It is possible the wide-field configuration of the electrode array led to non-specific increases in overall excitability of the spinal cord, which makes it difficult to contrast blood pressure changes driven by sympathetic activation with those from lower limb muscle contraction. However, blood pressure modulation can occur without muscle activation (Harkema et al., 2018) suggesting activation of autonomic networks as a contributing factor. To better understand cardiovascular effects of rostral and caudal stimulation configurations, and selective activation of sympathetic networks, more studies comparing responses by configuration will be needed.

In other studies, in individuals with peripheral vascular disease, scES is used to minimize pain and increase blood flow to diseased limbs with minimal changes to blood pressure (Huber et al., 2000). scES at the lower thoracic and lumbar levels causes vasodilation and increased blood flow to the lower limbs (Tallis et al., 1983; Jacobs et al., 1988), possibly from inhibition of nociceptive afferents (Jacobs et al., 1988) or modulation of autonomic activity (i.e., sympathetic inhibition) (Huber et al., 2000). Contrary to this, in persons implanted at T5-T6 for treatment of neuropathic pain, there were no significant changes to blood pressure with and without stimulation. However, Schultz and colleagues found that scES significantly increased blood pressure during a cold pressor test (a test used to investigate sympathetic nervous system activity) compared with a cold pressor test alone (Schultz et al., 2007). However, the same cold pressor test and stimulation at the levels of T1-T2 generated no such response. The differences between our results and other studies could be the etiology of the diseases themselves: peripheral vascular disease results from increased sympathetic activity originating in the spinal cord, while neuropathic pain is thought to result, in part, from nociceptive afferents increasing sympathetic nervous system activity (Huber et al., 2000; Campbell and Meyer, 2006). Application of scES is thus modulating an overactive sympathetic nervous system in these diseases, whereas in SCI, scES increases excitability of motor and autonomic networks that are largely silent.

Our contrasting results emphasize not only the complexity of the spinal cord, but the complexity of scES itself. We have found previously that stimulation parameters are crucial determinants of the extent to which scES modulates spinal circuits and restores function (Harkema et al., 2011; Angeli et al., 2014; Rejc et al., 2015, 2017a,b). In this study, scES parameters (electrode configuration, voltage, and frequency) were selected specifically to elicit motor activity. Because the electrode was configured to facilitate neuromuscular activity, it seems cardiovascular effects of scES reported herein are upon a combination of neuromuscular-induced changes in venous return and excitation of local sympathetic efferents. It is therefore possible different scES parameters could be used to specifically activate autonomic structures and lead to more precise modulation of cardiovascular outcomes. In addition, autonomic effects should be considered when deciding on scES for motor activity especially in those with cardiovascular deficits.

## Conclusion

Lumbosacral scES configured to facilitate motor function can acutely increase arterial blood pressure in individuals with SCI who suffer cardiovascular dysregulation without extensive training. This effect could be used to mitigate orthostatic intolerance and maintain blood pressure while standing. Also, importantly, in those with SCI with normal blood pressure scES does not elicit hypertension while using stimulation for improving motor behavior. The observation that epidural stimulation intended to facilitate motor activity also has the potential to modulate blood pressure opens a new avenue of research to better understand how to manage unstable blood pressure while pursuing physical rehabilitation in the SCI population.

### Limitations and future directions

In order to develop scES as a therapeutic option for treatment of systemic and orthostatic hypotension, greater understanding of the mechanism behind its effects will be necessary. This will require more detailed investigation of the mechanisms of cardiovascular dysfunction associated with SCI using an appropriate sample size. This study was limited to seven individuals and should be repeated in a larger number of individuals with varied levels and severity of injury. Additionally, individuals were grouped by cardiovascular outcomes, and differences in function due to injury may add variability but were not accounted for. Analyses of mechanisms, including the role of motor activity, autonomic network activation, and hormone changes in larger cohorts designed specifically to understand effects of stimulation on cardiovascular function are warranted. Effects of scES on the respiratory system, related to motor activity and cardiovascular deficits, also should be understood. Further, assessment of different configurations (anode and cathode selection, frequency, amplitude, and pulse width) on cardiovascular outcomes are needed. Better understanding the mechanism of action may lead to development of scES into a therapeutic strategy for management of unstable blood pressure in persons with SCI.

## Author contributions

The concept and design of this study were developed by SH, AK, CA, SA, and YC. SA, BL, MP, CA, AO, AK, and SH were involved in data acquisition, analysis, and interpretation. The manuscript draft written by SA, BL, MP, AO, and SH was critically revised by all authors.

### Conflict of interest statement

MP has the following financial relationships to disclose: (1) Listed faculty for University of Minnesota Educational Partnership with Medtronic Inc., Minneapolis, MN, (2) Grant/Research support from: Medtronic Inc., Boston Scientific, Advanced Neuromodulation Systems, Inc.,—St. Jude Medical, St. Jude Medical. SH has the following financial relationships to disclose: (1) NeuroRecovery Education and PowerNeuroRecovery. The other authors declare that the research was conducted in the absence of any commercial or financial relationships that could be construed as a potential conflict of interest.

## Abbreviations

AIS: American spinal injury Impairment Scale
ECG: electrocardiogram
EMG: electromyography
scES: Spinal Cord Epidural Stimulation
SCI: Spinal Cord Injury.
